# Evaluation of Risk and Prognosis Factors of Acute Kidney Injury in Patients With HELLP Syndrome During Pregnancy

**DOI:** 10.3389/fphys.2021.650826

**Published:** 2021-03-15

**Authors:** Lijuan Wang, Dongjie Tang, Haijun Zhao, Mingfeng Lian

**Affiliations:** ^1^Department of Emergency, Women and Children’s Hospital, School of Medicine, Xiamen University, Xiamen, China; ^2^Department of Hematology, The First Affiliated Hospital of Xiamen University and Institute of Hematology, School of Medicine, Xiamen University, Xiamen, China

**Keywords:** acute kidney injury, haemolysis elevated liver enzymes low platelet count, prognosis, pregnancy, mortality

## Abstract

Hemolysis, elevated liver enzymes, and low platelet count (HELLP) syndrome complicated with acute renal failure (AKI) is an important cause of maternal mortality and morbidity. The present retrospective study aims to identify risk and prognosis factors that are associated with AKI in patients with HELLP syndrome during pregnancy. A total of 110 pregnant HELLP patients with or without AKI from an 8-year period were studied. 65 of the patients were diagnosed with AKI based on the Kidney Disease Outcomes Quality Initiative criteria. Levels of the lowest hemoglobin and highest serum creatinine and bleeding incidence were identified as independent risk factors for AKI onset. Infection and serum creatinine level were identified as independent risk factors for maternal mortality. In addition, we also found that these factors were significantly different in AKI patients of different severity. The overall complete recovery rate was 67.7% (44 out of 65) for patients with AKI. The overall death rate was 4.5% (5 out of 110), where one of the patient was from the non-AKI group and the other four were from the AKI group. Our results provide valuable indications for clinical doctors during their diagnosis, treatment, and monitoring of recovery status in HELLP patients complicated with AKI.

## Introduction

Hemolysis, elevated liver enzymes, and low platelet count (HELLP) syndrome is a rare, but serious condition arising during pregnancy that occurs in 0.2–0.8% of all pregnancies (Abildgaard and Heimdal, 2013). The incidence rate rises to 10–20% for women with preeclampsia (Karumanchi et al., 2005) and to as high as 27.6% for women with eclampsia (Vigil-De Gracia et al., 2015). As a result, a stunning increase in the incidence rate of maternal and perinatal mortality and morbidity was observed in these patients (Sibai et al., 1993; Erdemoglu et al., 2010; Gedik et al., 2017). HELLP syndrome was initially described by Weinstein (1982) and its diagnosis is based on the laboratory analysis of microangiopathic hemolytic anemia, increased levels of liver enzymes, and thrombocytopenia in patients demonstrating symptoms of preeclampsia (Gasem et al., 2009).

Acute kidney injury (AKI) is considered a rare complication of pregnancy (Grunfeld and Pertuiset, 1987; Stratta et al., 1996). However, it is also known as a severe complication of HELLP syndrome, with reported AKI incidence rates between 7.7 and 60% in HELLP patients (Sibai et al., 1993; Martinez de Ita et al., 1998; Abraham et al., 2001; Gul et al., 2004; Erdemoglu et al., 2010; Gedik et al., 2017; Huang and Chen, 2017). In addition, HELLP syndrome is observed to be an important cause of AKI during pregnancy, leading to 15–65% of the total cases (Randeree et al., 1995; Selcuk et al., 2000; Abraham et al., 2001; Drakeley et al., 2002; Gul et al., 2004). Despite extensive studies discussing HELLP syndrome itself, only a few studies have focused on the predicting factors for the onset of AKI in patients with HELLP syndrome (Selcuk et al., 2000; Gul et al., 2004; Huang and Chen, 2017; Ye et al., 2019). The present retrospective study aims to evaluate the risk and prognostic factors of AKI in patients with HELLP syndrome during pregnancy.

## Materials and Methods

### Patients

The present retrospective study was based on an inpatient database at the Women and Children’s Hospital of Xiamen University from May 2012 to December 2020. We screened for all pregnant patients diagnosed with HELLP syndrome. Only patients who were diagnosed with HELLP at the time of admission, but not patients who developed HELLP after admission were included in the present study. Patients experiencing chronic kidney disease (CKD) and/or diabetes mellitus were not included in the study. CKD was defined as previously described, including declined renal function characterized by a glomerular filtration rate less than 60 ml/min/1.73 m^2^, proteinuria, and/or hematuria for at least 3 months (Levey et al., 2003). All lab values displayed in Table 1 were measured at the time of hospital admission. The study was approved by the ethical committee of the Women and Children’s Hospital of Xiamen University. Consent was obtained from all participating patients.

**TABLE 1:** Comparison of the variables between HELLP patients with and without AKI.

Variables	Non-AKI patients (*n* = 45)	AKI patients (*n* = 65)	*p*-value
Age (years)	30.22 ± 4.65	31.26 ± 4.80	0.26
History of hypertension	5 (11.1%)	8 (12.3%)	0.848
Multipara	19 (42.2%)	28 (43.1%)	0.929
Cesarean delivery	40 (88.9%)	61 (93.8%)	0.351
Preeclampsia	32 (71.1%)	50 (76.9%)	0.491
Eclampsia	9 (20%)	15 (23.1%)	0.701
No. of patient with bleeding > 400 ml	6 (13.3%)	20 (30.8%)	0.034
Estimated bleeding volume (ml)	363.0 ± 109.2	401.4 ± 158.8	0.162
Infection	3 (6.7%)	12 (18.5%)	0.076
Perinatal death	14 (31.1%)	21 (32.3%)	0.895
Maternal death	1 (2.2%)	4 (6.2%)	0.362
SBP ≥ 160 mmHg	22 (48.9%)	43 (66.2%)	0.07
DBP ≥ 110 mmHg	21 (46.7%)	40 (61.5%)	0.123
Onset time of HELLP (weeks)	31.29 ± 3.27	31.71 ± 3.51	0.523
Lowest platelet count (×10^9^ cell/L)	42.1 ± 14.07	53.62 ± 15.21	<0.001
Lowest hemoglobin (g/L)	80.29 ± 13.98	88.92 ± 11.47	<0.001
LDH (U/L)	1034.20 ± 359.78	768.96 ± 128.59	<0.001
AST (U/L)	140.19 ± 41.29	244.78 ± 107.64	<0.001
ALT (U/L)	190.97 ± 67.30	193.58 ± 62.80	0.838
Bilirubin (μmol/L)	46.42 ± 34.20	25.09 ± 9.54	<0.001
Serum creatinine (μmol/L)	71.92 ± 8.93	210.89 ± 129.34	<0.001

*SBP, systolic blood pressure; DBP, diastolic blood pressure; LDH, lactate dehydrogenase; AST, aspartate transaminase; and ALT, alanine transaminase.*

### Diagnosis

Diagnosis of HELLP syndrome was based on previously published criteria (Sibai et al., 1993; Ye et al., 2019), including microangiopathic hemolytic anemia based on detection in a peripheral blood smear; elevated lactate dehydrogenase (LDH) or bilirubin levels greater than 600 U/L or 20.5 μmol/L, respectively; liver dysfunction indicated by elevated aspartate transaminase (AST) levels greater than 70 U/L; and a platelet count less than 100 × 10^9^/L.

Diagnosis of AKI was based on the Kidney Disease Outcomes Quality Initiative (KDOQI) criteria (National Kidney Foundation, 2003), including an elevation of serum creatinine for more than 26.5 μmol/L within a 48-h window or more than 1.5 times higher than the baseline level detected during the past 7 days; a urine volume less than 0.5 mL/kg/h for 6 h. AKI was further classified into three stages according to the KDOQI criteria. Stage 1 was defined as an increase in serum creatinine for more than 26.5 μmol/L or 1.5–1.9 times the 7-day baseline level or a urine output less than 0.5 mL/kg/h for 6–12 h. Stage 2 was defined as an increase in serum creatinine 2.0–2.9 times the 7-day baseline level or a urine output less than 0.5 mL/kg/h for over 12 h. Stage 3 was defined as an increase in serum creatinine over 353.6 μmol/L or more than three times the 7-day baseline level or initiation of renal replacement therapy or a urine output less than 0.5 mL/kg/h for over 24 h or anuria for more than 12 h.

Preeclampsia was diagnosed as the onset of hypertension (blood pressure over 140/90 mmHg) and proteinuria (urinal protein level over 0.3 *g* every 24 h) after 20 weeks of gestation. If hypertension was detected prior to hospital admission, preeclampsia was diagnosed by proteinuria or terminal organ dysfunction after 20 weeks of gestation. Eclampsia was diagnosed when seizures was observed in preeclampsia patients.

### Follow-Up

The recovery of all included patients was followed for at least 1 year post-partum or until death. Recovery status was recorded at 1 month, 3 months, 6 months, and 1 year time points. Complete recovery of AKI was achieved when serum creatinine levels dropped to a normal range (85–97 μmol/L). Partial recovery was recorded when improved renal function was observed, although the serum creatinine levels remained higher than the normal range. Infection-positive patients were identified based on clinical manifestations and positive microbiological results. Infection was observed both before and after the onset of AKI.

### Statistics

Statistical analysis was performed with the SPSS software (version 16.0, IBM, Armonk, NY, United States). Kolmogorov–Smirnov test was used to check the normality of the data. Variables that are normally distributed were analyzed using the independent sample *t* test or one-way ANOVA. Variables that are not normally distributed were analyzed using the Mann–Whitney *U* test. Categorical variables were analyzed using the χ^2^ test.

Univariate and multivariate logistic regression models were performed on the variables that were identified to be significantly different between patients with and without AKI. Predictive factors for maternal mortality were identified by logistic regression analysis performed on all included patients. A *p*-value of less than 0.05 was considered statistical significant for all analyses.

## Results

### Patient Characteristics

A total of 110 patients diagnosed with HELLP syndrome administered at our institution were included in the present study. AKI was detected in 65 (59.1%) of them. Women with AKI had significantly higher incidence of bleeding >400 mL (*P* = 0.034), the lowest platelet counts (*P* < 0.001), lowest hemoglobin (*P* < 0.001), as well as levels of LDH, AST, bilirubin, and serum creatinine (*P* < 0.001 for all; Table 1). The cut-off of 400 ml for bleeding was chosen according to the standard used by a previous study (Ye et al., 2019). However, the average estimated bleeding volume for non-AKI and AKI patients were 363.0 ± 109.2 ml and 401.4 ± 158.8 ml, respectively, which were not statistically significant.

In term of infection, three cases were found in patients without AKI (6.7%), which were all pulmonary infection. Twelve cases were found in patients with AKI (18.5%), including pulmonary infection (*n* = 6), uterine cavity infection (*n* = 3), and gastrointestinal infection (*n* = 3). 32 (71.1%) and 50 (76.9%) patients in the non-AKI and AKI groups experienced preeclampsia, respectively (Table 1). Among them, nine (20%) and 15 (23.1%) developed into eclampsia (Table 1). Fourteen (31.1%) and 21 (32.3%) perinatal deaths were found in the non-AKI and AKI groups, respectively (Table 1).

A total of five death cases (4.5%) were reported in all participating patients, one patient was from the non-AKI group and the other four were from the AKI group (Table 1). The patient without AKI passed away due to manifested HELLP syndrome, but with a live birth. The four patients with AKI passed away due to postpartum hemorrhage (*n* = 2), primary pulmonary hypertension (*n* = 1), and heart failure (*n* = 1).

Next, we further divided the AKI patients into three stages according to the KDOQI criteria. There were 28 patients at stage 1 (43.1%), 19 patients at stage 2 (29.2%), and 18 patients at stage 3 (27.7%). Significant differences in the levels of lowest platelet count, lowest hemoglobin, LDH, bilirubin, and serum creatinine (*P* < 0.001 for all) were found among the patients at the three different stages (Table 2). For the patients in the stage 3 AKI group, 10 of them had to be treated with renal replacement therapy, among which eight patients experienced anuria for more than 1 week.

**TABLE 2:** Comparison of the variables between HELLP patients with different severities of AKI.

Variables	AKI stage 1 (*n* = 28)	AKI stage 2 (*n* = 19)	AKI stage 3 (*n* = 18)	*p*-value
Age (years)	31.58 ± 4.86	30.82 ± 4.99	31.25 ± 4.59	0.854
History of hypertension	4 (14.3%)	2 (10.5%)	2 (11.1%)	0.913
Multipara	15 (53.6%)	7 (36.8%)	6 (33.3%)	0.324
Cesarean delivery	25 (89.3%)	19 (100%)	18 (100%)	0.845
Eclampsia	7 (25%)	4 (21.1%)	4 (22.2%)	0.947
Bleeding (>400 ml)	11 (39.3%)	4 (21.1%)	5 (27.8%)	0.392
Infection	6 (21.4%)	3 (15.8%)	3 (16.7%)	0.864
Perinatal death	5 (17.9%)	3 (15.8%)	4 (22.2%)	0.875
Maternal death	1 (3.6%)	1 (5.3%)	2 (11.1%)	0.573
SBP ≥ 160 mmHg	18 (64.3%)	13 (68.4%)	12 (66.75)	0.956
DBP ≥ 110 mmHg	17 (60.7%)	10 (52.6%)	13 (72.2%)	0.469
Onset time of HELLP (weeks)	31.81 ± 3.24	30.86 ± 3.40	30.75 ± 3.17	0.485
Lowest platelet count (×10^9^ cell/L)	50.42 ± 13.12	34.80 ± 10.98	34 ± 8.88	<0.001
Lowest hemoglobin (g/L)	89.59 ± 12.05	72.96 ± 10.30	69.73 ± 8.06	<0.001
LDH (U/L)	835.67 ± 222.79	1115.69 ± 305.19	1397.48 ± 38.89	<0.001
AST (U/L)	132.49 ± 42.31	154.16 ± 39.19	134.48 ± 38.89	0.148
ALT (U/L)	201.89 ± 60.77	191.82 ± 70.45	161.17 ± 74.12	0.207
Bilirubin (μmol/L)	29.07 ± 13.32	49.06 ± 32.02	86.42 ± 41.88	<0.001
Serum creatinine (μmol/L)	116.18 ± 19.86	236.60 ± 110.61	408.41 ± 61.13	<0.001

*SBP, systolic blood pressure; DBP, diastolic blood pressure; LDH, lactate dehydrogenase; and AST, aspartate transaminase.*

### Predicting Factors for AKI Onset in HELLP Syndrome

We picked the variables that were found to be significantly different between the non-AKI and AKI patients (Table 1) and performed univariate logistic regression analysis to assess the association of AKI onset with HELLP syndrome. We found that all six variables were independently associated with AKI onset in patients with HELLP syndrome (Table 3). In terms of bleeding, it is worth noting that only the number of patients with bleeding greater than 400 ml, but not the estimated bleeding volume was associated with AKI onset (Table 3). Then, we included all these variables in multivariate logistic regression analysis and found that bleeding, lowest hemoglobin, and highest serum creatinine levels remained independent risk factors for AKI onset (Table 3). We also performed univariate logistic regression analysis to assess the association of history of hypertension, preeclampsia, and perinatal death with stage of AKI and revealed no significant relations for all the variables (Table 4).

**TABLE 3:** Univariate and multivariate logistic regression analyses of factors associated with AKI.

Variables	Univariate analysis		Multivariate analysis	
	Odds ratio (95% CI)	*p*-value	Odds ratio (95% CI)	*p*-value
No. of patient with bleeding > 400 ml	0.1162 (0.0417, 0.3236)	0.0002	0.1657 (0.0632, 0.3147)	0.013
Estimated bleeding volume (ml)	0.9354 (0.7745, 1.1966)	0.2522		
Lowest platelet count (×10^9^ cell/L)	0.9491 (0.9227. 0.9762)	0.0003		0.122
Lowest hemoglobin (g/L)	0.9509 (0.9216, 0.9811)	0.0016	1.0365 (0.9145, 1.1023)	0.019
LDH (U/L)	1.0046 (1.0021, 1.0070)	0.0002		0.241
AST (U/L)	0.9825 (0.9752, 0.9898)	0.0001		0.254
Bilirubin (μmol/L)	1.0595 (1.0248, 1.0954)	0.0007		0.178
Serum creatinine (μmol/L)	1.0346 (1.0169, 1.0785)	0.0003	1.3548 (1.0254, 1.6358)	0.009

*LDH, lactate dehydrogenase and AST, aspartate transaminase.*

**TABLE 4:** Univariate logistic regression analyses of factors associated with AKI stage.

Variables	Univariate analysis	
	Odds ratio (95% CI)	*p*-value
History of hypertension	1.254 (1.014–1.436)	0.589
Preeclampsia	1.148 (0.956–1.342)	0.634
Perinatal death	1.025 (0.874–1.315)	0.745

### Predicting Factors for Maternal Death in HELLP Syndrome

Next, we re-grouped the patient data into survivors and non-survivors, and compared the variables between the two groups. Infection (*P* < 0.001), perinatal death (*P* < 0.001), systolic blood pressure (SBP) ≥ 160 mmHg (*P* < 0.001), diastolic blood pressure (DBP) ≥ 110 mmHg (*P* < 0.001), lowest hemoglobin (*P* = 0.013), LDH (*P* = 0.023), bilirubin (*P* = 0.002), and serum creatinine (*P* < 0.001) were found to be significantly different between the two patient groups (Table 5). Then, we performed univariate logistic regression analysis on these variables and identified all of them as independent risk factors for maternal death (Table 6). In addition, when including all these variables in a multivariate regression model, infection and serum creatinine level were still identified as independent risk factors (Table 6).

**TABLE 5:** Comparison of the variables between survivors and non-survivors.

Variables	Survivors (*n* = 105)	Non-survivors (*n* = 5)	*p*-value
Age (years)	30.87 ± 4.77	30.2 ± 4.6	0.26
History of hypertension	12 (11.4%)	1 (20%)	0.562
Multipara	45 (42.9%)	2 (40%)	0.9
Cesarean delivery	97 (92.4%)	4 (80%)	0.324
Eclampsia	23 (21.9%)	1 (20%)	0.92
Bleeding (>400 ml)	25 (23.8%)	1 (20%)	0.845
Infection	11 (10.5%)	4 (80%)	<0.001
Perinatal death	30 (28.6%)	5 (100%)	<0.001
SBP ≥ 160 mmHg	60 (57.1%)	5 (100%)	<0.001
DBP ≥ 110 mmHg	56 (53.3%)	5 (100%)	<0.001
Onset time of HELLP (weeks)	31.37 ± 3.36	33.4 ± 3.05	0.189
Lowest platelet count (×10^9^ cell/L)	47.07 ± 15.39	41.45 ± 19.95	0.433
Lowest hemoglobin (g/L)	84.52 ± 13.53	69.18 ± 5.28	0.013
LDH (U/L)	910.81 ± 295.81	1238.27 ± 563.62	0.023
AST (U/L)	184.74 ± 92.81	146.01 ± 41.33	0.357
ALT (U/L)	193.34 ± 64.96	164.67 ± 71.98	0.339
Bilirubin (μmol/L)	35.87 ± 27.56	76.11 ± 32.38	0.002
Serum creatinine (μmol/L)	142.61 ± 105.33	393.31 ± 183.04	<0.001

*SBP, systolic blood pressure; DBP, diastolic blood pressure; LDH, lactate dehydrogenase; and AST, aspartate transaminase.*

**TABLE 6:** Univariate and multivariate logistic regression analyses of factors associated with survival.

Variables	Univariate analysis		Multivariate analysis	
	Odds ratio (95% CI)	*p*-value	Odds ratio (95% CI)	*p*-value
Infection	0.0293 (0.0030, 0.2856)	0.0024	0.03548 (0.0134, 0.0548)	0.005
Perinatal death		<0.001		0.431
SBP ≥ 160 mmHg		<0.001		0.243
DBP ≥ 110 mmHg		<0.001		0.279
Lowest hemoglobin (g/L)	1.1121 1.0140, 1.2197)	0.0242		0.436
LDH (U/L)	0.9977 (0.9955, 0.9999)	0.039		0.397
Bilirubin (μmol/L)	0.9742 (0.9549, 0.9939)	0.0106		0.134
Serum creatinine (μmol/L)	0.9879 (0.9803, 0.9957)	0.0023	1.0564 (0.8965, 1.1354)	0.002

*SBP, systolic blood pressure; DBP, diastolic blood pressure; and LDH, lactate dehydrogenase.*

### Recovery Outcome

Patients with AKI were followed for 1 year to document their recovery outcome (Table 7). In total, 44 of the patients achieved complete recovery within the 1-year follow-up period. For the 21 partially recovered patients, 17 of them had a serum creatinine level higher than 120 μmol/L and four of them still had SBP and DBP higher than 160 and 110 mmHg, respectively. As expected, the mean values of serum creatinine after the 1-year follow-up period for the AKI and non-AKI groups were 114.5 ± 40.2 and 90.4 ± 11.4 μmol/L, respectively, with statistical significance (*P* < 0.001).

**TABLE 7:** Comparison of the recovery outcomes between HELLP patients with and without AKI.

Recovery outcome	AKI patients (*n* = 65)
Complete recovery	
Within 1 month	31 (47.7%)
Within 3 months	7 (10.8%)
Within 6 months	3 (4.6%)
Within 1 year	3 (4.6%)
Partial recovery	21 (32.3%)

## Discussion

Despite the decreasing incidence rate of pregnancy-related AKI, it remains a critical cause of maternal and fetal morbidity and mortality (Vigil-De Gracia et al., 2015). A number of previous studies have suggested that the HELLP syndrome is a primary cause of pregnancy-related AKI (Sibai et al., 1993; Martinez de Ita et al., 1998; Abraham et al., 2001; Gul et al., 2004; Erdemoglu et al., 2010; Gedik et al., 2017; Huang and Chen, 2017). In the present study, AKI developed in 65 out of 110 HELLP patients (59.1%), which is generally in line with two previous studies performed on Chinese populations that had a 48.1% (Ye et al., 2019) and 60% (Huang and Chen, 2017) AKI development rate, respectively. Given that many of the previous studies performed on the subject had rather limited sample sizes (Martinez de Ita et al., 1998; Erdemoglu et al., 2010; Gedik et al., 2017; Ye et al., 2019), the findings of our study will add confidence on the present view of diagnosis and treatment of HELLP and AKI patients, especially for the Chinese population. In three studies based on Turkish HELLP patient populations, 14 out of 126 (11%; Erdemoglu et al., 2010), 19 out of 77 (25%; Gedik et al., 2017), and 20 out of 132 (15%; Gul et al., 2004) patients developed AKI. In an American HELLP patient population, 7.7% of the patients developed acute renal failure (AKI; Sibai et al., 1993). Another recent study performed on the American population to determine the prevalence of AKI, placental abruption, and postpartum hemorrhage in patients with preeclampsia or HELLP syndrome revealed a 14.4% AKI developmental rate in HELLP patients (Novotny et al., 2020). In addition, 34 out of 173 (20%) patients developed AKI in a Mexican HELLP patient population (Martinez de Ita et al., 1998). Taken together, it seems that the Chinese HELLP patient population has a much higher tendency to develop AKI compared to other parts of the world. In fact, Chinese and white populations have been implicated as clinical correlates of the HELLP syndrome (Williams and Wilson, 1997). BMI and lifestyle might affect such prevalence since it has been shown to affect the prevalence of preeclampsia (Xiao et al., 2014). In addition, since the present study was carried out over a period of 8 years (2012 to 2020), the substantial improvement in healthcare for renal disease might also affect the observed outcome.

Hemolysis, elevated liver enzymes, and low platelet count syndrome often leads to severe maternal and neonatal consequences, with reported maternal mortality rates varying from 0 to 12% (Sibai et al., 1993; Abraham et al., 2001; Gul et al., 2004; Erdemoglu et al., 2010). When experiencing AKI on top of the HELLP syndrome, the maternal mortality rate increased from 12 to 34% (Sibai et al., 1993; Randeree et al., 1995; Martinez de Ita et al., 1998). Microcirculatory blood flow and capillary density have also been implicated in patients with HELLP syndrome by increasing the sensitivity of the kidney to ischemia (Ospina-Tascon et al., 2017). Here, we found a maternal mortality rate of 3.6% (4 out of 110) in HELLP patients complicated with AKI, which is much lower than the previously reported range. Very few studies have investigated the risk and prognosis factors for AKI onset and maternal mortality in HELLP patients. In the present study, we found that lowest hemoglobin, LDH, bilirubin, and serum creatinine were independent risk factors for both parameters. In addition, all four factors were also found to be significantly different in AKI patients of different severity. These results provide a strong indication that these variables should be closely monitored during the diagnosis, treatment, and recovery periods of HELLP patients with AKI.

Previous studies have reported perinatal mortality rates between 7 to 26% and 26 to 34% in HELLP syndrome alone (Gul et al., 2004; Erdemoglu et al., 2010) and HELLP syndrome plus AKI (Sibai et al., 1993; Gul et al., 2004), respectively. A recent meta-analysis that included 11 cohort studies revealed that HELLP syndrome is associated with a relatively higher risk of AKI during pregnancy, fetal mortality, and maternal death (Liu et al., 2020). In the present study, we found these two values to be 31.1% (14 out of 45) and 32.3% (35 out of 65) for these two groups of patients, where the former is slightly higher than the reported value range and the latter falls within the known range.

So far, the most effective treatment against HELLP syndrome remains to be the termination of pregnancy, and most HELLP patients start to recover within the first 24 to 48 h after delivery (Martin, 2013). Alternatively, postpartum plasma exchange was reported to be effective for HELLP syndrome and was recommended to be the treatment option of consideration for all HELLP patients who demonstrate no significant improvement in AST and platelet levels within 24 to 48 h after delivery (Simetka et al., 2015). Previous studies have reported a favorable recovery outcome of patients with HELLP syndrome complicated with AKI, where most of them were discharged from the hospital without any significant renal impairment (Sibai et al., 1993; Selcuk et al., 2000; Gul et al., 2004). Animal models of HELLP+AKI have also been developed to study the underlying cellular and molecular mechanisms of the two pathological conditions (Wallace et al., 2018). In the present study, we revealed a complete recovery rate of 86.7% (39 out of 45) in non-AKI HELLP patients, but only 67.7% (44 out of 65) in HELLP patients with AKI. The length of follow-up time does not seem to be a factor that accounts for such a discrepancy, since a study focusing on long-term renal function after HELLP syndrome revealed no association between the HELLP syndrome and long-term renal complications after a 5-year follow-up period (Jacquemyn et al., 2004). The worse than before recovery rates could be due to the difference in the analyzed patient population. Indeed, the overall complete recovery rate for the present study was 75.5% (83 out of 110), which is similar to a previous study performed on the Chinese population (Ye et al., 2019).

It is known that HELLP often overlaps with atypical hemolytic uremic syndrome (aHUS) and thrombotic thrombocytopenic purpura (TTP) during the perinatal period, leading to challenging diagnosis, especially with the onset of AKI. In the present study, the diagnosis of HELLP syndrome was based on the detection of microangiopathic hemolytic anemia, elevated lactate dehydrogenase or bilirubin levels, liver dysfunction, and platelet count. Levels of a disintegrin and metalloproteinase with a thrombospondin type 1 motif, member 13 (ADMADS-13), and HUS-related complement system activation and regulation were not included in the present study due to the retrospective nature of the study. However, the easily recognizable symptoms of liver dysfunction and the rapidly recovered levels of AST, ALT, and hemolysis after delivery provide supportive evidence for the diagnosis of HELLP rather than aHUS or TTP.

The present study is limited by its retrospective nature so that we cannot determine the causal impact of the assessed variables on AKI onset and maternal mortality in HELLP patients. Also, there may be a lack of information on how the proposed lab values may have changed during the 1-year postpartum follow-up period. In addition, since it is a single institution study, the number of patients included is also rather limited. This may result in an inadequately powered study. To maximize the number of patients in this study, we had to collect data over an 8-year period, during which a substantial improvement in healthcare occurred and might have affected the accuracy of the analysis. However, similar previous studies covered similar [7.5 years in Huang and Chen (2017)] or even longer [15 years in Ye et al. (2019)] data periods, indicating that the length of the study period should have its validity. Therefore, future prospective studies with a larger patient quantity are needed to further verify the present findings. Moreover, there were only five death cases in the present study, which may cause potential inaccuracies in the data comparison between the survivor and the non-survivor groups. Furthermore, the uneven distribution of the patient number within the three AKI groups might also lead to an inaccurate conclusion, although the *p*-values were all less than 0.001 for all characteristics that exhibited statistical differences among the three groups.

In summary, we have identified a number of risk and prognosis factors that are closely associated with the onset of AKI (levels of the lowest hemoglobin and highest serum creatinine and bleeding incidence) and maternal mortality rate (infection and serum creatinine level) in HELLP patients, which provide indications for clinical doctors on the diagnosis, treatment, and recovery of such patients. Our findings also further support the present view of diagnosis and treatment of HELLP and AKI patients, especially for the Chinese population.

## Data Availability Statement

The original contributions presented in the study are included in the article/supplementary material, further inquiries can be directed to the corresponding author/s.

## Ethics Statement

The studies involving human participants were reviewed and approved by Women and Children’s Hospital of Xiamen University. The ethics committee waived the requirement of written informed consent for participation.

## Author Contributions

LW, DT, and HZ performed the research. ML designed the study and wrote the manuscript. All authors contributed to the article and approved the submitted version.

## Conflict of Interest

The authors declare that the research was conducted in the absence of any commercial or financial relationships that could be construed as a potential conflict of interest.
